# Glycoengineering of Therapeutic Antibodies with Small Molecule Inhibitors

**DOI:** 10.3390/antib10040044

**Published:** 2021-11-04

**Authors:** Shasha Li, Alex J. McCraw, Richard A. Gardner, Daniel I.R. Spencer, Sophia N. Karagiannis, Gerd K. Wagner

**Affiliations:** 1 Medical Biology Centre, School of Pharmacy, Queen’s University Belfast, 97 Lisburn Road, Belfast BT9 7BL, UK; Shasha.Li@qub.ac.uk; 2 St. John’s Institute of Dermatology, School of Basic & Medical Biosciences, King’s College London, 9th Floor Tower Wing, Guy’s Hospital, London SE1 9RT, UK; alexa.mccraw@kcl.ac.uk (A.J.M.); sophia.karagiannis@kcl.ac.uk (S.N.K.); 3 Ludger, Ltd., Culham Science Centre, Abingdon, Oxfordshire OX14 3EB, UK; richard.gardner@ludger.com (R.A.G.); daniel.spencer@ludger.com (D.I.R.S.); 4 Breast Cancer Now Research Unit, Guy’s Cancer Centre, School of Cancer & Pharmaceutical Sciences, King’s College London, London SE1 9RT, UK

**Keywords:** antibody, immunoglobulin, glycan, glycoengineering, glycosylation, glycoform, inhibitor, chemical tools

## Abstract

Monoclonal antibodies (mAbs) are one of the cornerstones of modern medicine, across an increasing range of therapeutic areas. All therapeutic mAbs are glycoproteins, i.e., their polypeptide chain is decorated with glycans, oligosaccharides of extraordinary structural diversity. The presence, absence, and composition of these glycans can have a profound effect on the pharmacodynamic and pharmacokinetic profile of individual mAbs. Approaches for the glycoengineering of therapeutic mAbs—the manipulation and optimisation of mAb glycan structures—are therefore of great interest from a technological, therapeutic, and regulatory perspective. In this review, we provide a brief introduction to the effects of glycosylation on the biological and pharmacological functions of the five classes of immunoglobulins (IgG, IgE, IgA, IgM and IgD) that form the backbone of all current clinical and experimental mAbs, including an overview of common mAb expression systems. We review selected examples for the use of small molecule inhibitors of glycan biosynthesis for mAb glycoengineering, we discuss the potential advantages and challenges of this approach, and we outline potential future applications. The main aim of the review is to showcase the expanding chemical toolbox that is becoming available for mAb glycoengineering to the biology and biotechnology community.

## 1. Introduction

Since their introduction as therapeutic agents some 45 years ago, monoclonal antibodies (mAbs) have become one of the most important modalities in modern medicine. Not only are they a cornerstone of the clinical management of several haematopoietic and tissue cancers, the therapeutic use of mAbs has also been extended to a variety of non-oncological diseases including migraine [1], cardiovascular disease [2] and asthma [3]. As of 2019, over 80 antibody-based therapeutics have received regulatory approval in Europe and/or the US across a variety of disease types, of which 15 are indicated for the management of solid tumours [4,5]. Antibodies have rapidly become amongst the top-selling drugs, with the global antibody market expected to generate revenue in the range of USD 300 billion by 2025 [6].

Immunoglobulin G (IgG) is the most abundant antibody class in human serum, representing approximately 80% of circulating immunoglobulins [7,8]. IgG is also the most common structural scaffold for recombinant therapeutic antibodies, although other immunoglobulins such as Immunoglobulin E (IgE) and Immunoglobulin A (IgA) are increasingly being explored for such applications [9,10]. A shared feature across all immunoglobulins is that they are glycoproteins: i.e., their polypeptide chain is decorated with glycans, oligosaccharides of extraordinary structural diversity. The presence, absence, and composition of these glycans has a profound effect on mAb structure and function, including effector functions, effector cell engagement, overall efficacy, antigenicity, solubility, stability and safety. The manipulation of mAb glycan structures—glycoengineering—is therefore an exciting and promising strategy for the optimisation of these critical parameters for improved therapeutic performance.

In this review, we highlight the use of small molecule inhibitors for glycoengineering of therapeutic antibodies. We focus on small molecules that have already been used, or may be suitable, for applications in mAb glycoengineering, discuss properties that are relevant for such applications, and briefly introduce their target enzymes. We also provide a molecular perspective on the role of glycans for mAb function. This review is complementary to an excellent recent publication covering other glycoengineering approaches—not discussed here—such as genetic engineering of cell lines, chemo-enzymatic glycan remodelling, and glycoengineering for site-specific antibody–drug conjugation [11]. Our main aim is to showcase the expanding chemical toolbox for mAb glycoengineering to the biology and biotechnology community.

## 2. Immunoglobulins and Their Glycans

### 2.1. Immunoglobulins and Their Functions

Immunoglobulins are heterodimeric glycoproteins that are structurally comprised of heavy and light chains, which in turn can be functionally separated into variable domains (Fab region—antigen binding) and constant domains (Fc region—effector functions) [8,12]. Part of the humoral immune system, human antibodies are categorised into five immunoglobulin (Ig) classes: IgG, IgE, IgA, IgM, and IgD; with IgG and IgA further subdivided into subclasses (isotypes), IgG1-4 and IgA1-2 (Table 1). Generally, each isotype possesses distinct structural properties and biological roles, as well as both unique and overlapping functions. The immune cell types engaged, and the effector mechanisms elicited, vary between isotypes.

Mechanisms underlying antibody function can be either Fab-mediated—the recognition of specific antigens triggering downstream responses on target cells—or Fc-mediated—interactions with immune effector cells leading to immune cell or complement activation and immune clearance of pathogens [22]. Direct mechanisms of antibody function include neutralisation of pathogens and toxins, in which antibodies bind their corresponding antigens to prevent interactions with host cell receptors, and opsonisation whereby pathogens are bound and marked for phagocytosis by antibodies. Otherwise, antibodies can drive removal of pathogenic or infectious agents indirectly.

The first of these indirect mechanisms is complement-dependent cytotoxicity (CDC), which occurs through immunoglobulin-mediated activation of the complement cascade, leading to upregulation of inflammatory mediators, opsonisation of immune complexes, and direct destruction of targets via membrane attack complexes [8]. The second, recruitment of effector cells, drives cell-mediated effects such as antibody-dependent cell-mediated cytotoxicity (ADCC) and antibody-dependent cellular phagocytosis (ADCP), both involving recruitment of effector cells including natural killer (NK) cells, macrophages, and monocytes to either cytotoxically kill target cells, or phagocytose opsonised pathogens [23]. IgG, the most abundant antibody class, is particularly effective at driving CDC and ADCC [7,8], although individual IgG isotypes have distinct biological roles and functional profiles (Table 1). IgG-class antibodies elicit their functions through engaging different cognate Fcγ receptors, with these interactions driving different effector responses (Table 2).

### 2.2. Immunoglobulins and Their Glycans

The biological and therapeutic functions of antibodies are strongly influenced by their glycans. Glycosylation is a common post-translational modification of immunoglobulins, of which there are two distinct categories—*N*-linked glycans, attached to Asn, and *O*-linked, on Ser/Thr [29] (this review focuses predominantly on *N*-glycosylation). While there appears to be no consensus sequence for *O*-glycosylation, *N*-glycosylation typically occurs at Asn–X–Ser/Thr sites (where X = any amino acid). All human immunoglobulins possess between one (IgG1, IgG2 and IgG4) and seven (IgE) *N*-glycosylation sites (Table 1). *N*-Glycosylation occurs during protein synthesis and is required for native expression and correct protein folding, and the resulting glycans directly influence, in many cases, immunoglobulin structure and function [30]. Ig glycosylation influences both antibody structure and function, with differential glycosylation patterns directly affecting Ig expression, conformation, pharmacokinetics, binding to Fc receptors, and recognition of antigens. Glycan composition can therefore impact the biological functions of antibody isotypes, from antigen processing and presentation to effector cell engagement, and modify the nature and strength of downstream immune responses [30,31]. A molecular understanding of Ig glycans is therefore critical for the development of mAbs with enhanced effector functions and optimized therapeutic attributes.

**Figure 1:**
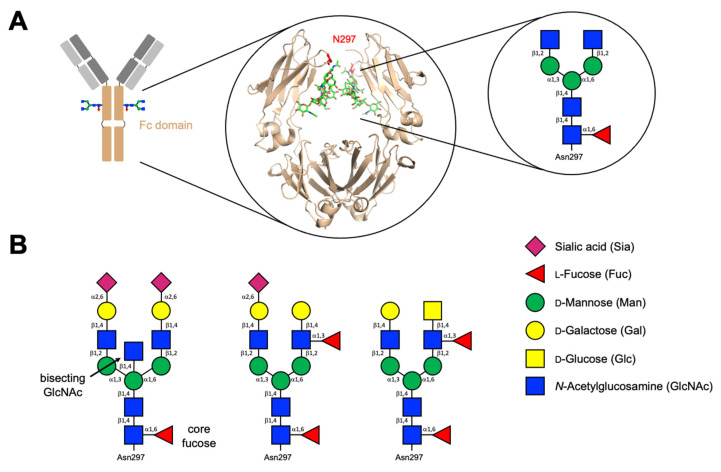
(**A**) Cartoon representation and 3D structure of the Fc domain of a typical IgG and its Fc *N*-glycan. The crystal structure is taken from PDB 4Q7D, colour code: wheat, protein backbone; green, *N*-glycan; red, glycosylation site N297. (**B**) Common biantennary complex-type *N*-glycans found on mature glycoproteins.

***Immunoglobulin G (IgG)***. Previous studies concerning the role of glycans in modulating the functions and structures of human immunoglobulins have focused on the human IgG subclasses and their respective Fcγ receptors (Table 1). The Fc domain of IgG possesses a single core glycan at the *N*-glycosylation site Asn297 (N297) on each heavy chain (Figure 1), the presence of which is required for binding to Fcγ receptors [32], although around 20% of IgG antibodies will contain additional glycans in the Fab regions. These additional sugars are poorly understood but are known to show differential glycosylation in health and disease states [32,33]. N297 oligosaccharides are typically biantennary complex-type glycans decorated with core L-fucose (Fuc), bisecting *N*-acetyl d-glucosamine (GlcNAc), terminal d-galactose (Gal), and terminal sialic acid (Sia) (Figure 1) [34,35]. This core glycan is conserved across all IgG subclasses and glycoforms; however, terminal sugars vary even in healthy individuals, and upwards of 30 glycoforms have been identified for the N297 glycan [32]. Alongside asymmetrical glycosylation between heavy chains within the same IgG antibody, this creates a diverse array of IgG glycosylation variants.

Effects of variable sugar residues on IgG function have been extensively studied. d-Galactose appears to influence IgG structure and in turn, the type of effector function that can be driven—agalactosylated IgG antibodies have been reported to be more efficient at driving complement-dependent cytotoxicity, whilst hyper-galactosylated antibodies were shown to enhance the ADCC response via increased FcγRIIIa binding [32,36]. Sialic acid, meanwhile, is linked to anti-inflammatory responses: terminal *α*2,6-sialylation of the IgG Fc can greatly influence the anti-inflammatory activity of human intravenous immunoglobulin (IVIG) [34,35] and stimulate upregulation of inhibitory FcγRIIB [37]. Additionally, evidence suggests that increasing IgG Fc-sialylation decreases its ability to drive CDC [37]. In mice, Fc-sialylation is suggested to shift the balance from Type 1 FcγR interactions (classical Ig receptors; Table 2) to Type 2 FcγR (non-classical receptors such as DC-SIGN and CD23) [38]; however, this has been confirmed to not occur in humans [39].

L-Fucose is perhaps the most important IgG terminal glycan sugar, with multiple studies pointing towards a role for fucose in destabilising the interactions between IgG Fc and FcγRIIIa glycans, with binding subsequently enhanced through stabilising carbohydrate-carbohydrate interactions in the absence of L-fucose leading to increased ADCC capability [40]. Such observations have prompted the development of afucosylated mAbs for cancer therapy, discussed below. The presence or absence of bisecting GlcNAc (b-GlcNAc) residues also appears to influence the ADCC response, however, this may simply be due to afucosylation, since the presence of β-GlcNAc impedes access of fucosyltransferases to the core glycan [32]. Similarly, high mannose levels linked to enhanced immunogenicity of IgG and improved ADCC may also be due to lack of L-fucose [41], although high-mannose-engineered antibodies have shown decreased binding to C1q and consequently decreased ability to drive CDC [42,43].

Our understanding of the above glycan structure/function relationships is derived mainly from studies with IgG1, whilst the exact involvement of glycans in the functions of other IgG isotypes remains to be fully elucidated [40]. Nonetheless, there is substantial evidence that, for IgG at least, differential antibody glycosylation does influence effector function responses elicited. Fine-tuning the IgG glycosylation pattern therefore offers an opportunity for the generation of antibodies specialised towards desirable functional profiles.

In the context of the ongoing COVID-19 pandemic, it may be of particular interest that the severity of SARS-CoV-2 infections has been correlated with particular IgG glycoforms. Thus, both an increase in IgG afucosylation [44,45] and a decrease in bisecting *N*-acetylglucosamine (GlcNAc) and galactosylation were observed in severe COVID-19 patients [46]. A decrease in sialylation also contributes to enhanced inflammatory activity by ADCC regulation [47]. The reduced sialylation and galactosylation may play a role in COVID-19 pathogenesis via the activation of the lectin-initiated alternative complement pathway.

***Immunoglobulin A (IgA)***. IgA, the second most prevalent circulatory human antibody class and most abundant in secretions, is heavily glycosylated (Table 1), with its glycans forming up to 10% of its molecular weight. IgA has two isoforms—IgA1 and IgA2—which differ in their glycosylation profiles, and differences in glycans are also reported between the serum and secretory forms [9]. IgA glycans predominantly consist of a biantennary mannosyl chitobiose core with triantennary structures [9]. The exact levels of terminal sugar residues appear to vary and, for IgA1, the presence of additional O-linked glycans at the hinge region introduces further diversity. Fab region glycosylation on IgA has also been reported, with these glycans suggested to be more heavily sialylated than their Fc counterparts [9,31].

The roles of IgA glycosylation are most well-documented for the secretory form (sIgA), with the secretory component (SC) known to contain seven *N*-linked sites in addition to the 2 sites per heavy chain of the core antibody [9,31]. Alongside O-linked glycans present in the hinge, which protect the antibody from bacterial proteolysis, these *N*-linked glycans facilitate ligand binding and represent binding sites for lectins and adhesins, which in turn can facilitate cognate Fc receptor (FcαRI)-mediated effector signalling [31]. Whilst a reduction in terminal d-galactose residues is reported to be an underlying factor of IgA nephropathy, triggering reduced clearance of IgA from the circulation leading to nephropathy onset [8], terminal sugar residues overall appear not to heavily influence IgA binding. Some glycoengineering studies with recombinant IgA have indicated that there may indeed be no difference between IgA glycoforms in FcαRI binding affinity, and that instead FcαRI glycosylation may be the determinant of IgA-FcαRI binding affinity [9]. Other, more recent work has suggested, in contrast, that differences in functional profiles between the IgA isotypes may be partly attributable to differential glycosylation profiles [16]. These seemingly conflicting results highlight the fact that we are only just beginning to understand the precise role of *N*-glycans for mAb activity.

***Other immunoglobulins***. For the remaining human antibody classes, the picture is even less clear. For human IgM and IgD, relatively little is known about the involvement of glycosylation in function. Mutations preventing glycosylation at N354 in IgD are known to disrupt IgD production and expression on the surface of B cells, indicating that these glycans may be essential for expression and stability [8]. A similar phenomenon is observed in IgM, with complete abolishment of *N*-glycosylation shown to disrupt both secretion and function [13], although generally this observation holds true across most human immunoglobulins and is not unique. IgM carries 5 documented *N*-linked glycan sites on each heavy chain, but little is known about glycan involvement in IgM function. However, it is presumed they may in some way influence interactions between IgM and other immune effector cells [13,48].

The matter of IgE glycosylation, meanwhile, is still subject to much controversy. IgE is the most heavily glycosylated human immunoglobulin class with seven *N*-linked glycosylation sites and glycans representing 12% of its total molecular weight (Table 1) [49]. Whilst it is known that native glycosylation is required for IgE secretion [50], whether or not further processing of core glycans is an absolute requirement for IgE structure and function is subject to debate. The N275 glycan, considered equivalent to the IgG N297 glycan, is the sole IgE glycan with an extensively documented functional role, having been reported as essential for binding to the cognate high-affinity receptor FcεRI and influencing relevant downstream effector functions, including allergic responses such as anaphylaxis [51]. No consensus has yet been reached on whether factors such as terminal sugar residues and the presence or absence of other site-specific glycans impact the structure and/or functions of IgE, although recent insights have now suggested sialic acid may regulate the allergic response [52].

### 2.3. mAb Glycoengineering

To fully understand the complex roles of *N*-glycans for immunoglobulin biology, the generation of immunoglobulins with defined glycan structures is indispensable. This can be achieved by glycoengineering—the manipulation of protein glycan structures. Glycoengineering represents a powerful tool to optimise the pharmacodynamic and pharmacokinetic properties of therapeutic mAbs, and to deliver homogeneous mAb glycoforms during production: an important consideration from a regulatory and manufacturing perspective.

A famous example for the successful glycoengineering of mAbs is the enhanced ADCC of afucosylated antibodies. Engineered afucosylated IgG1 antibodies bind with higher affinity to FcγRIIIa expressed on immune effector cells such as NK cells and monocytes/macrophages, thus enhancing cell-mediated effector functions such as ADCC [53], and two afucosylated mAbs have been thus far approved for cancer treatment. Obinutuzumab (Gazyva) is a humanised anti-CD20 monoclonal antibody for the treatment of Chronic lymphocytic leukaemia (2013) [54] and follicular lymphoma (2016, 2017) [55], whilst mogamulizumab (Poteligeo) is a humanized, monoclonal antibody targeting the CC chemokine receptor 4 (CCR4), which gained FDA approval in 2018 for treatment of relapsed or refractory mycosis fungoides and Sézary syndrome [56].

Both obinutuzumab and mogamulizumab were developed by engineering cell lines to reduce the amount of fucose attached to the antibody during production. While such genetic manipulation of glycan biosynthesis has remained the predominant method for glycoengineering to date, a range of alternative approaches have emerged in recent years, including gene silencing via RNA interference (RNAi) technology [57], gene editing with CRISPR/Cas-9 [58], and in vitro glycan remodeling using endoglycosidases and glycosyltransferases [34,59].

Another attractive alternative is the addition of small molecule inhibitors of glycosylation to the culture medium during protein production [60]. In the next chapter, we review selected small molecule inhibitors that have either been used successfully for the glycoengineering of mAbs already, or that may be suitable for such applications. We also consider the advantages and challenges of using small molecule inhibitors, and briefly introduce relevant molecular targets and mAb expression systems.

## 3. Small Molecule Inhibitors for the Glycoengineering of Monoclonal Antibodies

### 3.1. N-Glycan Biosynthesis in Eukaryotes

**Figure 2:**
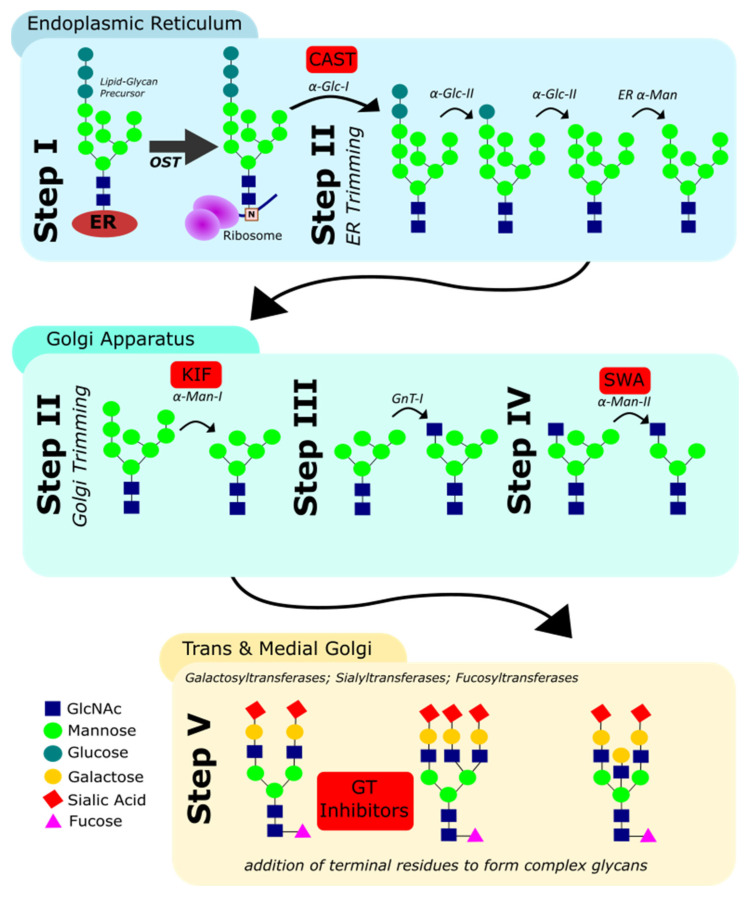
Key steps during eukaryotic *N*-glycan biosynthesis. Red boxes indicate inhibitors of individual enzymes (CAST: castanospermine; KIF: kifunensine; SWA: swainsonine; see Section 3.4).

Small molecule inhibitors of protein *N*-glycosylation usually act by inhibiting one or more enzymes involved in *N*-glycan biosynthesis. In eukaryotes, *N*-glycan biosynthesis occurs concomitant to protein synthesis via the sequential interplay of multiple glycosidases and glycosyltransferases in the endoplasmic reticulum (ER) and Golgi. Glycosyltransferases are anabolic enzymes that catalyse the formation of glycosidic bonds by transferring a mono- or oligosaccharide from a glycosyl donor to an acceptor molecule, while glycosidases are catabolic enzymes that catalyse the cleavage of these glycosidic bonds. Key steps during the biosynthesis of eukaryotic *N*-glycans include (Figure 2):

(i) The *en bloc* transfer of an oligosaccharide to the asparagine residue in N-X-ST sequons of the nascent polypeptide by the oligosaccharyl transferase (OST);
(ii) The trimming of terminal d-glucose (Glc) and d-mannose (Man) residues by glucosidases and mannosidases in the ER and cis-Golgi;
(iii) The addition of a *N*-acetyl d-glucosamine (GlcNAc) residue onto the intermediate Man_5_GlcNAc_2_ structure;
(iv) The further removal of Man residues by mannosidases in the medial-Golgi; and finally;
(v) The elaboration of the resulting hexasaccharide by addition of GlcNAc, d-galactose (Gal), L-fucose (Fuc) and sialic acid (Sia) residues. These final steps, including the addition of terminal residues, are catalysed by different glycosyltransferases in the medial- and trans-Golgi.

The individual glycosidases and glycosyltransferases involved in this process usually display exquisite substrate specificity, including for the nature of the sugar, the position on the sugar where transfer/cleavage occurs (regiospecificity), and the stereochemistry of the glycosidic bond that is being formed or cleaved. All glycosyltransferases involved in eukaryotic *N*-glycan biosynthesis use sugar-nucleotides such as GDP-L-fucose, UDP-d-galactose and CMP-sialic acid as their monosaccharide donors, with the exception of OST, whose donor is a lipid-linked oligosaccharide.

### 3.2. mAb Expression Systems

Species-dependent variations of the general *N*-glycan biosynthetic pathway pose practical challenges for the generation of therapeutic antibodies. Non-human expression systems, mainly Chinese Hamster Ovary (CHO) cells, followed closely by the rodent lines NS0 and Sp2/0, remain predominant for the production of biological therapeutics in the pharmaceutical industry [61]. However, non-humanised glycans on therapeutic antibodies can drive severe, even fatal, anaphylactic reactions in humans [62]. A well-known example is α-Gal, the mammalian galactose-α1,3-galactose linkage not found in higher apes or humans, that has been linked to hypersensitivity responses to the EGFR-specific mAb cetuximab [63,64]. Analysis of cetuximab, which is typically produced in Sp2/0 cells [63], revealed that of the 21 glycan structures characterised, around 30% carry α-Gal residues [64].

Plant-based expression systems such as Nicotiana benthamiana are becoming increasingly popular but can introduce potentially immunogenic plant glycans including α1,3-fructose, and β1,2-xylose [62]. Whilst hypersensitivity responses towards these glycans seems to be less common, data suggests they present a similar degree of immunogenicity to mammalian glycans: supporting this, the plant-derived therapeutic taliglucerase alfa has a similar hypersensitivity incidence amongst patients to cetuximab [65]. Whilst insect cell expression systems can efficiently produce recombinant proteins with complex glycan patterns; their glycosylation, in addition to potential immunogenicity, shows significant variation from human counterparts [66]. Insect glycans resemble trimmed *N*-glycan precursors with high levels or mannose or paucimannose with no terminal sialic acid or galactose and may not necessarily result in the same functional or structural profile as a glycoprotein derived from a mammalian system [66].

While the use of human cell lines may represent an obvious solution, care must also be taken when using human-compatible cell lines. Although a clear benefit of human or humanised cell lines is human-compatible post-translational modifications (PTMs) similar to those on native human proteins; drawbacks include risk of human-specific viral contamination, inconsistent PTMs [66], and in the case of humanised cell lines such as CHO, inactive genes—such as the α1-3-galactosyltransferase responsible for addition of α-Gal—becoming transiently or even permanently activated during the course of transfection and expression, leading to inclusion of unwanted glycans [67].

Human embryonic kidney cell (HEK293) expression systems, for example, can be used for production of therapeutic mAbs, ensuring a humanised glycan profile; however, it has been noted that these cells can introduce a high degree of heterogeneity in glycan structures [68]. Analysis of monoclonal IgE generated in a HEK932 expression system found as many as 30 glycoforms [68,69]. Whilst such heterogeneity may not be problematic for classes such as IgE, the functional profile of which so far seems unaffected by glycan content [70], for IgG, this could introduce unwanted effects. As discussed above, presence of fucose decreases interactions with the IgG receptor FcγRIIIa, resulting in decreased ADCC [40], and increased levels of mannose, another sugar residue of interest, is linked to more rapid serum clearance, potentially requiring higher dosages to overcome [71]. Asides from innate effects on glycan content, cell media too must be carefully considered as presence of nonhuman glycoproteins in culture media can lead to scavenging and subsequent inclusion in expressed glycoproteins of nonhuman glycans [67]. Taken together, these examples illustrate the challenges arising from species-dependent variations in *N*-glycan biosynthesis, some of which can be addressed with small molecule inhibitors.

### 3.3. Small Molecule Inhibitors: Advantages and Challenges

An alternative to the genetic disruption of glycan biosynthesis is the use of small molecule enzyme inhibitors. This approach offers several practical advantages: it is cost effective and operationally simple, as small molecules can be readily added to established culture protocols. Moreover, a given small molecule inhibitor can be used in conjunction with any number of different cell lines and genetic backgrounds, and the extent of the intervention can be modulated, in contrast to genetic knock-outs, by adjusting the inhibitor concentration.

On the other hand, the effective use of small molecules in cell culture also faces a number of challenges: lack of potency, poor cell uptake, and limited chemical and/or enzymatic stability of inhibitors can all result in modest activity in cells. Both on- and off-target toxicity may preclude the application of certain inhibitors in cells altogether, and target promiscuity can complicate the generation of defined glycoforms and the delineation of structure/function relationships.

Critical features of the ideal small molecule inhibitor for glycoengineering therefore include:

- High potency against its molecular target;
- Target specificity (or known off-target profile);
- Good cellular uptake and activity in cell culture;
- Chemical and enzymatic stability in the culture medium;
- No cell toxicity;
- No detrimental effect on antibody yield.
- While many inhibitors of carbohydrate-active enzymes such as glycosidases and glycosyltransferases have been reported, the number of inhibitors with suitable properties for applications in cell culture is still limited.

### 3.4. Inhibitors of Carbohydrate-Active Enzymes for mAb Glycoengineering

Most small molecule inhibitors that have been used for glycoengineering target either individual glycosidases or glycosyltransferases directly, or, in the case of glycosyltransferases, reduce the availability of the sugar-nucleotide donor substrate. In the following sections, we review selected examples of small molecule inhibitors suitable for use in mAb glycoengineering.

#### 3.4.1. Glycosidase Inhibitors

**Figure 3:**
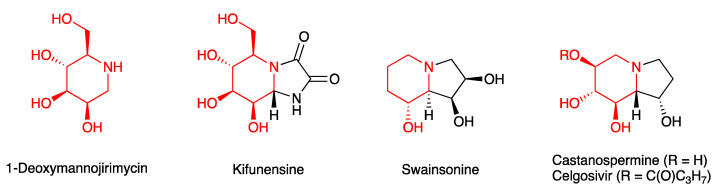
Glycosidase inhibitors 1-deoxymannojirimycin, kifunensine, swainsonine, and castanospermine, and its prodrug celgosivir. The iminosugar motif is shown in red.

Glycosidase inhibitors have been developed for a wide range of applications, from drug discovery to biotechnology [72]. Many classical glycosidase inhibitors are derived from natural products, such as iminosugars and alkaloids (Figure 3). These inhibitors frequently contain a structural element that can mimic the substrate or transition state of the glycosidase reaction, although there is usually no strict correlation between stereochemistry and enzyme target (α-glucosidase vs. α-mannosidase). A number of glycosidase inhibitors have been investigated as molecular tools for the glycoengineering of mAbs and other recombinant therapeutic glycoproteins.

Kifunensine (Figure 3), an alkaloid from the actinobacterium *Kitasatosporia kifunense*, is a potent inhibitor of α-mannosidase I which is located in the cis-Golgi (Figure 2). Inhibition of this enzyme in cell culture, e.g., with the mannosidase inhibitors kifunensine or 1-deoxymannojirimycin, leads to the accumulation of high mannose glycoproteins bearing Man_7–9_GlcNAc_2_ oligosaccharides. A current manufacturing process of the recombinant glycoprotein therapeutic velaglucerase alpha in HT1080 fibrosarcoma cells exploits this property of kifunensine, as a high mannose content improves mannose-receptor mediated uptake into macrophages [73], which in this case is desired.

Kifunensine has also been used successfully for the generation of afucosylated rituximab in the *Nicotiana benthamiana* transient expression platform [74]. The afucosylated antibody showed a 14-fold increase in ADCC activity against the lymphoma cell line Wil2-S compared to standard rituximab [74]. The wider application of kifunensine for the generation of afucosylated mAbs may be complicated by the concomitant increase in high mannose glycans, which often leads to faster clearance of mAbs in vivo [71].

Kifunensine and the related glycosidase inhibitors swainsonine and castanospermine (Figure 3) have also been used to modulate the glycan profile of the engineered, chimeric antibody EG2-hFc in CHO cells [75]. Castanospermine blocks the removal of the first (and therefore all subsequent) d-glucose residue(s) during the initial trimming step in the ER, whilst swainsonine is an inhibitor of α-mannosidase II in the medial Golgi (Figure 2). This enzyme is required for the removal of two outer mannose residues from the nascent *N*-glycan, leading to the generation of a biantennary structure that is recognised as a substrate by downstream glycosyltransferases.

The effects on *N*-glycosylation of EG2-hFc were different for each of the three inhibitors. Kifunensine increased the amount of high mannose glycans, castanospermine resulted in high mannose with attached glucose glycans, and swainsonine allowed for fucosylation of hybrid structures with and without sialylation. For comparison, all three inhibitors were also used for the glycoengineering of the full sized, humanized IgG1 antibody DP-12 under the same conditions [75]. The presence of hybrid glycan structures decreased binding to FcγRI to a similar extent for both mAbs, while complete removal of the *N*-glycan had a much stronger effect on DP-12 binding compared to EG2-hFc [75].

Although beyond the scope of this review, it is interesting to note that glycosidase inhibitors have recently also attracted considerable interest as potential drug candidates against COVID-19, reflecting the importance of antibody and antigen glycosylation for SARS-CoV-2 infections. For example, Yang and co-workers have shown that the glycoforms of the SARS-CoV-2 spike protein control the rates of viral entry into human HEK293T cells, and that an 85–90% reduction of viral entry can be achieved with the α-mannosidase I inhibitor kifunensine [76]. Similarly, celgosivir, an ester prodrug of the α-glucosidase I inhibitor castanospermine, has shown promising activity towards SARS-CoV-2 *in vitro* [77].

#### 3.4.2. Fucosylation Inhibitors

**Figure 4:**
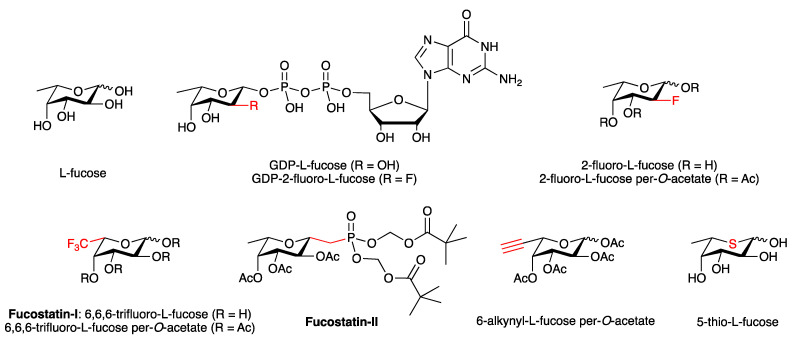
l-Fucose, the fucosyltransferase donor GDP-l-fucose, and fucosylation inhibitors discussed in the text. Relevant structural motifs are shown in red.

L-Fucose (Figure 4) is a 6-deoxyhexose that is typically found as a terminal residue on *N*-glycans (Figure 1). Fc core fucosylation adversely affects the ADCC functions of IgG antibodies, with the removal of core L-fucose therefore having clinical benefits for mAb therapy, as demonstrated by the success of the afucosylated mAbs obinutuzumab and mogamulizumab. While these antibodies were obtained from genetically engineered cell lines, there are also several chemical strategies for the generation of afucosylated or low-fucose mAbs with small molecules.

Fucosylation is a late-stage modification during glycan biosynthesis, which is catalyzed by fucosyltransferases in the Golgi apparatus (Figure 2). The common sugar-nucleotide donor of these fucosyltransferases is GDP-d-mannose, and structural analogues of GDP-d-mannose such as GDP-6-fluoro-d-mannose (Figure 4) have long been known as potent, competitive inhibitors in vitro [78]. The application of these donor analogues in cell culture can be complicated by the presence of the pyrophosphate linkage, which may limit cell uptake, due to its negative charge at physiological pH, and is also susceptible to chemical and enzymatic degradation.

These problems can be circumnavigated with metabolic inhibitors. Here, the general idea is that small modifications of the L-fucose scaffold, such as the replacement of an alcohol (OH) with a fluorine (F) substituent, which is similar in size to hydrogen, will be tolerated by the enzymes of the fucose salvage pathway. Thus, such fluorinated L-fucose analogues will be converted intracellularly into the corresponding, fluorinated GDP-L-fucose analogues, which in turn act as inhibitors of fucosylation. In a seminal study, Paulson and co-workers executed this strategy successfully to reduce cellular fucosylation in human HL-60 cells with 2-fluoro-L-fucose (Figure 4) [79].

Senter and co-workers subsequently employed the same inhibitor to produce almost completely defucosylated IgG1-based mAbs, not only in cell culture, but also *in vivo* in mice [80]. To maximise cell uptake, they also investigated the corresponding O-peracetylated derivative of 2-fluoro-L-fucose, which is readily deacetylated intracellularly and can therefore serve as a prodrug for its parent compound. Treatment of CHO cells in fucose-deficient media with either 2-fluoro-L-fucose or 2-fluoro-L-fucose per-*O*-acetate produced nonfucosylated antibodies with increased binding to human FcγRIIIA and enhanced antibody-dependent cellular cytotoxicity (ADCC), without significant impact on mAb production or cell viability. Moreover, p.o. administration of 2-fluoro-L-fucose in mice led to circulating IgGs that were almost completely devoid of fucose. This method was also used to produce an anti-CD40 antibody, SEA-CD40, which is currently undergoing phase I clinical trials [80].

Mechanistically, these fucose analogues inhibit fucosylation by depleting intracellular pools of GDP-L-fucose, the donor substrate of fucosyltransferases [80]. 2-Fluoro-L-fucose and related fucose derivatives are converted intracellularly into the corresponding guanosine diphosphate (GDP) conjugates, which in turn act as inhibitors of GDP-mannose 4,6-dehydratase (GMD), a key enzyme for the *de novo* biosynthesis of GDP-L-fucose. This mechanism of action is particularly effective in fucose-deficient CHO cell culture media, where the *de novo* pathway is the almost exclusive source of GDP-L-fucose.

A similar metabolic inhibition strategy has also been applied successfully with other fluorinated fucose analogues. Allen and co-workers demonstrated that protein fucosylation of anti-TRAIL 2 receptor (TR-2) IgG1 mAb and antimesothelin (MSLN) IgG1 mAb can be reduced in a dose-dependent manner by addition of 6,6,6-trifluorofucose per-*O*-acetate (Figure 4), a prodrug of 6,6,6-trifluorofucose (fucostatin-I), to the CHO cell culture medium [81]. Interestingly, fucostatin-I and its prodrug both displayed similar potency in cell culture, which suggests that fucostatin-I is taken up efficiently by CHO cells, presumably via an active fucose transport mechanism. Crystallographic studies with the corresponding GDP conjugate of fucostatin-I provided direct evidence for binding at GDP-mannose 4,6-dehydratase (GMD), which interestingly occurs not at the GDP-mannose substrate binding site, but at an allosteric binding site [81]. Subsequently, fucostatin-I was used by Goddard-Borger and coworkers to generate fucose-deficient IgG1 antibodies also in hybridoma cell lines [82].

A potential complication of metabolic inhibitors is their incorporation into the glycan of the therapeutic antibody, which is undesired. In CHO cells, this undesired incorporation was limited to about 0.5% of total glycans with 6,6,6-trifluorofucose per-*O*-acetate, and completely abolished with the fucophosphonate inhibitor fucostatin-II (Figure 4) [81]. Fucostatin-II contains a chemically and enzymatically stable bond at the anomeric centre, which precludes the transfer of the fucose moiety. Its inhibitory potency towards protein fucosylation (EC_50_ ~30 μM) is, however, slightly weaker than that of fucostatin-I (EC_50_ ~4 μM).

A range of other fluorinated fucose analogues have also been used as metabolic inhibitors of cellular fucosylation, including 5-thio-L-fucose [83] and 6-alkynyl-L-fucose [84]. In cells, 6-alkynyl-L-fucose is converted into GDP-6-alkynyl-L-fucose, which binds to the catalytic pocket of GDP-L-fucose synthetase, the enzyme downstream from GDP-mannose 4,6-dehydratase in the GDP-L-fucose *de novo* pathway. Although developed for other applications, in principle, these metabolic inhibitors of cellular fucosylation may also be applicable for mAb glycoengineering.

These examples illustrate the growing range of metabolic inhibitors that are now available to reduce fucosylation in cells and on proteins. Many of these inhibitors are already commercially available, which facilitates their application for mAb glycoengineering.

#### 3.4.3. Galactosyltransferase Inhibitors

d-Galactose residues in *N*-glycans affect both the complement-dependent and antibody-dependent cell-mediated cytotoxicity of antibodies as well as their *in vivo* half-life. These residues are installed by galactosyltransferases (GalTs) in the medial- or trans-Golgi (Figure 2), which catalyse the transfer of d-galactose from the sugar-nucleotide donor UDP-d-galactose (UDP-Gal) to carbohydrate acceptors during the final stages of *N*-glycan biosynthesis. Terminal d-galactose residues target glycoproteins to asialoglycoprotein receptors on the liver, thus limiting their *in vivo* half-life [85]. Reducing the amount of terminal d-galactose residues with GalT inhibitors is therefore a potential strategy to extend the *in vivo* half-life of mAbs and other therapeutic glycoproteins.

In *N*-glycans, d-galactose occurs most commonly in β1-4 linkages, e.g., as part of the *N*-acetyllactosamine (LacNAc) elongation motif in complex and hybrid *N*-glycans, and, to a lesser extent, in β1-3 linkages. These linkages are established with regio- and stereospecificity by the corresponding β1-4 and β1-3 GalTs. β1-4 GalTs catalyse the transfer of d-galactose from UDP-Gal specifically to GlcNAc acceptors. To date, seven isoforms of β1-4 GalT have been identified, almost all of which are expressed ubiquitously in mammals [86]. One of these isoforms, GalT4, is a major control point for glycan branching in *N*-linked glycosylation [87]. GalT inhibitors may therefore also be useful to control *N*-glycan branching.

**Figure 5:**
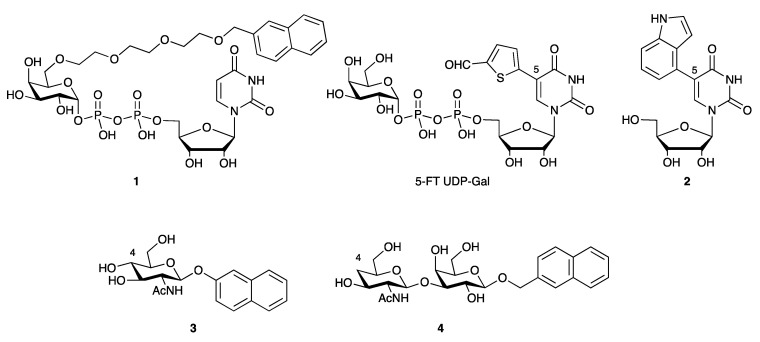
Galactosyltransferase inhibitors based on donor (**top**) or acceptor (**bottom**) substrate.

Many existing GalT inhibitors are substrate analogues that are based on the UDP-Gal donor, carbohydrate acceptor, or both [88]. Several donor analogues such as **1** and 5-FT UDP-Gal (Figure 5) have shown low micromolar or nanomolar activity against recombinant GalTs [89,90]. The key feature of 5-FT UDP-Gal and related inhibitors is the substituent in position 5, which blocks a conformational change in the enzyme active site that is crucial for catalysis [90,91,92]. Removal of the pyrophosphate and d-galactose moieties leads to a significant drop in activity, which can be compensated for in part by optimization of the 5-substituent [93].

Despite the presence of the charged pyrophosphate linkage, 5-FT UDP-Gal has shown activity in cells [94]. Thus, 5-FT UDP-Gal reduces the surface levels of the adhesion molecule P-selectin glycoprotein 1 (PSGL-1) in human monocytes [94]. These findings prompted us to also explore the applicability of this inhibitor to modulate galactosylation of mAbs. Initial results suggest that this may indeed be possible.

Acceptor analogues have also been reported as GalT inhibitors (Figure 5). Some classical acceptor-based inhibitors such as compound **3** retain substrate activity [95], which is not always reported in the literature and may complicate their application in cell culture. In contrast, the 4-deoxygenated disaccharide **4** is not recognized as an acceptor substrate by β1–4 GalT1, due of the absence of the 4-OH group [96]. Administered in the form of its per-O-acetylated prodrug, **4** reduces cell surface expression of the d-galactose-containing epitope Sialyl Lewis X on the surface of U937 human monocytic lymphoma-derived cells in a dose-dependent manner [96]. Although it was developed for a different application, due to its activity in cells, **4** may also be suitable for mAb glycoengineering.

#### 3.4.4. Sialyltransferase Inhibitors

Sialic acids (SAs) are a family of α-keto sugar acids containing a nine-carbon backbone. SAs are abundantly expressed as terminal residues at the non-reducing end of the carbohydrate moieties of mammalian cell surface glycoconjugates. Terminal SA residues also contribute to the regulation of antibody half-life and recycling. The installation of SAs during late-stage sialoglyan formation is catalysed by sialyltransferases (STs).

There are twenty human STs that are divided into four groups, ST3Gal I-VI, ST6Gal I-II, ST6GalNAc I-VI, and ST8Sia I-VI, based on their acceptor molecule and linkage types [97]. Each ST controls the synthesis of specific sialylated structures with unique biological roles. For example, Lewis antigen sialylation is controlled by ST3Gal III, IV, and VI, while β1 integrin sialylation is controlled by ST6Gal I [98].

**Figure 6:**
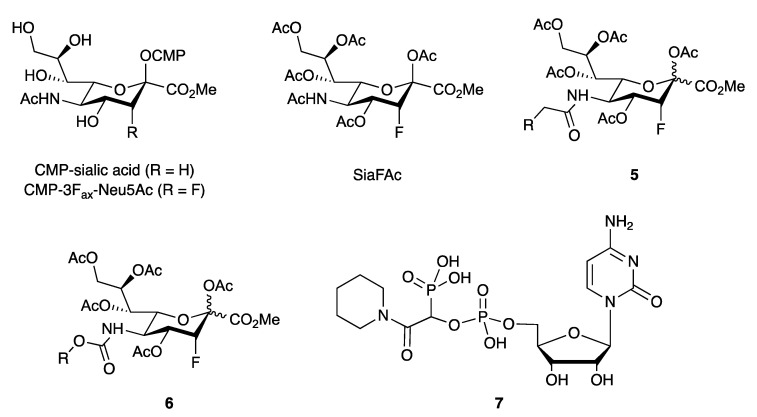
Sialyltransferase inhibitors.

Mammalian STs use CMP-sialic acid (also known as CMP-*N*-acetylneuraminic acid or CMP-Neu5Ac) as their donor substrate (Figure 6). First reported in 1999 [99], the fluorinated donor analog CMP-3F_ax_-Neu5Ac (Figure 6) is a potent ST inhibitor [78], but lacks activity in cells due to poor membrane penetration. In order to overcome this limitation, the peracetylated metabolic precursor SiaFAc was developed (Figure 6). Following passive diffusion across the cell membrane and intracellular deacetylation, SiaFAc is metabolically converted into the active CMP-3F_ax_-Neu5Ac [79]. CMP-3F_ax_-Neu5Ac potently reduces global sialylation in human HL-60 cells by inhibiting STs directly, as well as the *de novo* synthesis of the ST donor substrate CMP-sialic acid [79]. Heise et al. recently reported as series of SiaFAc analogues containing an amide (**5**) or carbamate (**6**) functionality at position C-5 (Figure 6). Some of these analogues showed considerably improved and prolonged inhibitory activity in multiple mouse and human cell lines [100]. Interestingly, introduction of a C-5 carbamate also resulted in more efficient metabolization into the active CMP conjugate [100].

The donor substrate CMP-sialic acid has also served as the template for the design of ST transition state inhibitors [101]. A recent example is the use of simple aliphatic or aromatic amides in conjunction with a phosphonate, to mimic the geometry and charge distribution of CMP-sialic acid in the ST transition state. While several of these inhibitors, exemplified by **7** (Figure 6), showed nanomolar activity against recombinant human ST6Gal-I, their activity in cells remains to be investigated [101].

#### 3.4.5. Non-Substrate-like Inhibitor Chemotypes

In addition to substrate-based inhibitors, a growing number of non-substrate-like inhibitor chemotypes for glycosyltransferases has been reported over the past few years [102]. Several of these inhibitors are uncharged, drug-like heterocycles with good activity in cells, which makes them interesting candidates for chemical glycoengineering.

**Figure 7:**
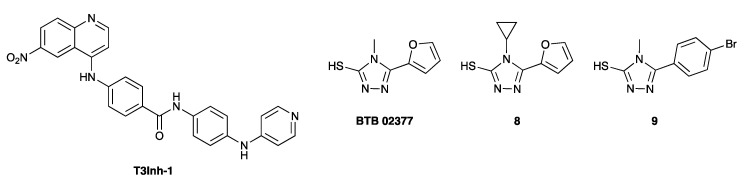
Non-substrate-like inhibitor chemotypes.

Typically, these inhibitors, such as the quinoline derivative T3Inh-1 (Figure 7), were identified from screening campaigns. T3Inh-1 is a potent and selective inhibitor of polypeptide *N*-acetylgalactosaminyltransferase 3 (ppGalNAc-T3), which catalyzes the first step in O-linked oligosaccharide biosynthesis [103]. The inhibitor reduces cancer cell invasiveness driven by upregulated ppGalNAc-T3 in cell culture, and also blocks ppGalNAc-T3-mediated glycan-masking of FGF23 in cells and mice with no toxicity [103]. T3Inh-1 may therefore also be an interesting tool to manipulate the O-glycan structures of mAbs.

Interestingly, 1,2,4-triazoles such as BTB 02377, **8** and **9** (Figure 7) have been reported as fucosyltransferase inhibitors in two independent recent studies [104,105]. This suggests that the 1,2,4-triazole-3-thiol motif might represent a common pharmacophore for fucosyltransferase inhibition, possibly through a covalent mode of action [104]. BTB 02377 is a potent inhibitor of FUT6, a human fucosyltransferase involved in the synthesis of sialyl Lewis^x^, with selectivity over FUT7 as well three different sialyltransferases [105]. These small, uncharged heterocycles are also very likely to be cell-permeable, although this has yet to be established.

## 4. Conclusions

Chemical glycoengineering is a promising approach for the optimization of mAbs and their therapeutic properties, and small molecule inhibitors of glycan biosynthesis are ideally suited for such applications. In contrast to alternative methods such as gene editing and knock-outs, the use of small molecule inhibitors is operationally extremely simple and applicable across different cell culture systems, making it highly attractive from a practical viewpoint.

While there are already successful examples for the chemical glycoengineering of mAbs, in particular with glycosidase inhibitors, there is also considerable untapped potential, especially in the area of glycosyltransferase inhibitors. To realise this potential, inhibitors with suitable properties for applications in cell culture are a prerequisite. Many existing glycosyltransferase inhibitors do not meet this requirement, although some may still be suitable, if used in conjunction with efficient cell delivery strategies.

Where can improved inhibitors come from? Two promising routes are the identification of new inhibitor chemotypes from screening [106], and, facilitated by the growing availability of structural information for glycosyltransferases, structure-based rational design [107]. Chemical glycoengineering may also take inspiration from other fields where glycosyltransferase inhibitors have long been sought after, such as anti-cancer and anti-bacterial drug discovery. Considering the scientific, therapeutic and, indeed, economic benefits any efficient method for the chemical glycoengineering of mAbs will almost certainly deliver, these are goals worth pursuing.

## Figures and Tables

**Table 1:** Key features of the nine human immunoglobulin (Ig) isotypes.

Ig Isotype	MW (kDa)	Biological Roles	*N*-Glycosylation Sites
IgG1	146	Most abundant IgG subclass forming the primary antibody response. Large role in response against viral infections; able to effectively drive complement-dependent cytotoxicity (CDC) [7]	N180 [13]
IgG2	146	Predominantly responds to glycans such as bacterial capsule polysaccharides. Roles in the bacterial immune response. Poor at driving CDC and antibody-dependent cell-mediated cytotoxicity (ADCC) [7]	N176 [13]
IgG3	170	Pro-inflammatory and highly potent mediator of effector functions such as CDC and ADCC. Large roles in the viral response. [7,14]	N227; N322 [13]
IgG4	146	Protective roles in allergy. Does not drive ADCC or CDC [7,15]	N177 [13]
IgA1	160 (serum) 385 (secretory)	Predominant serum IgA class. Mucosal defence. Less pro-inflammatory compared to IgA2 [16]	N144; N352 [13]
IgA2		Mucosal defence; cytokine production and NET formation via macrophages and neutrophils. Pro-inflammatory [16]	N47; N92; N131; N205; N327 [13]
IgE	196	Allergy and hypersensitivity; immune response against parasitic worms [8,17]	N21; N49; N99; N146; N252; N264; N275 [13]
IgM	190	Early immune response; B cell receptor [18,19]	N46; N209; N272; N279; N439 [13]
IgD	184	Involvement in activating B cells to produce antibodies; antimicrobial response [20,21]	N225; N316; N367 [13]

**Table 2:** Classical Fcγ receptors of IgG isotypes [7,24,25,26].

IgG Receptor	Specific Isotypes Engaged	Cell Expression	Immune Functions
FcγRI	IgG1; IgG3; IgG4	Monocytes/macrophages; Dendritic Cells (DCs); inducible expression on neutrophils and mast cells	Effector cell activation; phagocytosis [24]
FcγRIIa	N/A	Monocytes/macrophages Neutrophils; DCs; basophils; mast cells; eosinophils; platelets	Platelet activation and aggregation [27]; effector cell activation; phagocytosis; degranulation; ADCC [24]; antigen processing and presentation on DCs [28]
FcγRIIb	N/A	B cells; DCs; basophils; subsets of monocytes/ macrophages; subsets of neutrophils	Inhibition of effector activity [24]; limits DC maturation; opposes BCR signalling, and induces apoptosis to eliminate low affinity BCR B cells [28]
FcγRIIc	N/A	NK cells, monocytes/macrophages; neutrophils	Activating variant expressed in ~11% of individuals
FcγRIIIa	N/A	NK cells, monocytes/macrophages	Effector cell activation; ADCC; phagocytosis [24]
FcγRIIIb	IgG1; IgG3	Neutrophils; subsets of basophils	Unclear [24]
FcRN	IgG1	Endothelial and epithelial cells; monocytes/macrophages; neutrophils; DCs	Recycling of IgG in serum and protection from degradation; responsible for long serum half lives, transport of IgG across mucosal surfaces and placenta during pregnancy [7]
